# Progranulin serum levels in human kidney transplant recipients: A longitudinal study

**DOI:** 10.1371/journal.pone.0192959

**Published:** 2018-03-02

**Authors:** Bruna Bellincanta Nicoletto, Elis Forcellini Pedrollo, Larissa Salomoni Carpes, Natália Gomes Coloretti, Thaiana Cirino Krolikowski, Gabriela Corrêa Souza, Luiz Felipe Santos Gonçalves, Roberto Ceratti Manfro, Luis Henrique Canani

**Affiliations:** 1 Faculdade de Medicina, Universidade Federal do Rio Grande do Sul (UFRGS), Porto Alegre, Brazil; 2 Life Science Knowledge Area, Universidade de Caxias do Sul (UCS), Caxias do Sul, Brazil; 3 Department of Nutrition, Faculdade de Medicina, Universidade Federal do Rio Grande do Sul (UFRGS), Porto Alegre, Brazil; 4 Division of Nephrology, Hospital de Clínicas de Porto Alegre, Porto Alegre, Brazil; 5 Division of Endocrinology, Hospital de Clínicas de Porto Alegre, Porto Alegre, Brazil; Hospital Universitario de la Princesa, SPAIN

## Abstract

**Background:**

The adipokine progranulin has metabolic proprieties, playing a role in obesity and insulin resistance. Its levels seems to be dependent of renal function, since higher progranulin concentration is observed in patients with end-stage kidney disease. However, the effect of kidney transplantation on progranulin remains unknown.

**Objective:**

To assess the serum progranulin levels in kidney transplant recipients before and after kidney transplantation.

**Methods:**

Forty-six prospective kidney transplant recipients were included in this longitudinal study. They were evaluated before transplantation and at three and twelve months after transplantation. Clinical, anthropometric and laboratorial measurements were assessed. Progranulin was determined with enzyme-linked immunosorbent assays.

**Results:**

Serum progranulin significantly decreased in the early period after transplantation (from 72.78 ± 2.86 ng/mL before transplantation to 40.65 ± 1.49 ng/mL at three months; p<0.01) and increased at one year (53.15 ± 2.55 ng/mL; p<0.01 vs. three months), remaining significantly lower than before transplantation (p<0.01) (p_over time_<0.01). At one year after transplantation, there was a significant increase in body mass index, trunk fat and waist circumference compared to immediate period after transplantation. Progranulin was associated with waist circumference and fasting plasma glucose after adjusted for age, gender, study period, glomerular filtration rate, interleukin-6, high sensitivity C reactive protein and adiponectin.

**Conclusion:**

Progranulin serum levels are increased before transplantation and a reduction is observed in the early period after transplantation, possibly attributed to an improvement in renal function. At one year after transplantation, an increment in progranulin is observed, seems to be independent of glomerular filtration, and remained significantly lower than before transplantation.

## Introduction

Progranulin (PGRN), also known as proepithelin, granulin/epithelin precursor, or PC cell–derived growth factor, has emerged as a protein with growth factor-like properties, being involved in tissue remodeling, tumorigenesis and neurodegenerative diseases [1–4]. More recently, it has been associated with obesity and insulin resistance [5, 6]. PGRN is expressed in epithelial cells, immune cells, neurons and also in adipocytes [7], emerging as a novel adipokine with a function in glucose and insulin metabolisms [5, 6]. PGRN plays a role in adipose tissue, recruiting monocytes [8] and promoting interleukin-6 (IL-6) expression [5], which favors inflammation and insulin resistance. Previous studies have demonstrate that serum PGRN is increased in obesity and type 2 diabetes mellitus (T2DM) [8–10].

Elevated serum PGRN have also been observed in chronic kidney disease (CKD) [11, 12], and patients at grade 5 of CKD have increased PGRN levels [12]. PGRN follows a negative correlation with the eGFR in all grades of CKD, both in diabetic and non-diabetic populations [11–13].

Kidney transplantation is often followed by complications such as weight gain, increased body fat, dyslipidemia, metabolic syndrome and new onset diabetes after transplantation. These complications are possibly related to immunosuppressive therapy and changes in the metabolism [14–16]. Many adipokines have been studied in this context [17]. After kidney transplantation, their levels decrease, perhaps due to an improvement in eGFR [14, 18]; however, in the later period after transplantation, adipokine serum levels tend to increase [14].

Considering that the effect of kidney transplantation on PGRN serum levels has not been studied, and that this adipokine is related to renal function, we aimed to assess the effect of kidney transplant on serum PGRN concentration and its association with metabolic indexes.

## Materials and methods

### Design and patients

Forty-six patients who underwent kidney transplantation at the Hospital de Clínicas de Porto Alegre (Rio Grande do Sul, Brazil) between December 2014 and August 2015 were included in this longitudinal study. Kidney recipients were evaluated before transplantation and at three and twelve months after transplant. Data and blood samples at pre-transplant period were collected two days before surgery for the living donor recipients and immediately before surgery for the deceased donor organ recipients. A control group of 40 outpatients attending at the same hospital was included in the study. Controls were selected based on their renal function (eGFR between 30 and 90 mL/min/1.73m^2^), in order to match then to kidney recipients at twelve months (considering that eGFR at this moment would be comparable to controls). Moreover, groups were paired by age, gender and body mass index (BMI). Exclusion criteria were age below 18 years old, multiorgan transplantation, re-transplants, cancer, acute infections, Cushing’s disease, systemic lupus erythematosus disease, pregnancy, alcohol or drug abuse and kidney recipients who did not reach three months of transplantation with a functioning graft.

This study was approved by the Ethics Committee of Hospital de Clínicas de Porto Alegre and all subjects received adequate information about the study and gave their written informed consent.

### Clinical, anthropometric and laboratorial assessment

Demographic and clinical data were assessed using a standard questionnaire and review of medical registry, including the following variables: age, gender, ethnicity, primary kidney disease, dialysis modality and duration, donor type, immunosuppressive agents used, cumulative prednisone dose, arterial hypertension and previous or development of diabetes mellitus. Hypertension was defined by blood pressure ≥140/90 mmHg or antihypertensive medication use; while new onset diabetes after transplantation was defined by American Diabetes Association criteria [19, 20].

Anthropometric assessment consisted of weight, height, and waist circumference. Body weight (kg) and height (m) were evaluated in order to calculate BMI (kg/m^2^) [21]. Waist circumference was measured at the midpoint between the lowest rib and the iliac crest, using a flexible, inelastic measuring tape [22]. Body fat percentage (BF%) was assessed by two methods: 1) Tetrapolar bioelectric impedance device (Biodynamics 450; Biodynamics Corp Seattle, Washington, USA), using current of 800 microA and frequency of 50HkHz was performed at all study moments and groups; and 2) Dual-energy X-ray absorptiometry (DEXA) using a Lunar iDXA Densitometer and enCORE software (version 13,60,033; GE Healthcare, Madison, USA) was applied in control patients and in kidney recipients at three and twelve months after transplantation, since the pre-transplant logistics did not allow this analysis. Values obtained by two methods were positively correlated in the present study (BF% at 3 months r = 0.88, p<0.001 and 12 months r = 0.71, p<0.001). The measurements were performed with the patient fasting, without shoes, wearing light clothing, in a stable condition [23].

Blood samples were drawn after 12-hour overnight fasting and sera obtained by centrifugation were stored in duplicates at -80°C. PGRN, adiponectin (ADPN) and IL-6 were determined with enzyme-linked immunosorbent assays (all R&D Systems, Minneapolis, MN, USA). Of all samples, 36.6%, 31.3% and 28.3% were performed in duplicates for PGRN, ADPN and IL-6, respectively. The assay sensitivity and assay range was 0.54 ng/mL and 1.56-100 ng/mL for PGRN, 0.891 ng/mL and 3.9-250 ng/mL for ADPN and 0.7 pg/mL and 3.12-300 pg/mL for IL-6. For PGRN, ADPN and IL-6, the inter-assay coefficient was 8.1%, 7.2% and 3.0%, respectively.

Serum creatinine, high-sensitivity C reactive protein (hsCRP), insulin, fasting plasma glucose (FPG), total cholesterol, HDL-cholesterol and triglycerides were determined using standard local laboratory techniques. LDL-cholesterol was calculated using the Friedewald formula when triglyceride levels were lower than 400 mg/dL. The Homeostasis Model Assessment (HOMA) index was used to calculate the insulin resistance: HOMA = plasma insulin (μUI/mL) x fasting glucose (mmol/L) / 22.5. The eGFR was assessed by the Chronic Kidney Disease Epidemiology Collaboration (CKD-EPI) equation [24].

### Statistical analyses

The sample size calculation was based on a previous study that reported PGRN serum levels according to the 5 grades of CKD [12]. The PGRN concentration observed in patients at grade 5 was considered to be equivalent to pre-transplant period, while PGRN levels observed at grade 3 were considered to be similar to post-transplant period and control group, since an equivalent eGFR was expected. A difference of 25 ng/mL between moments and groups was admitted. Considering α = 0.05 and β = 0.10 errors and 20% losses after kidney transplantation, the estimated sample size was 35 individuals in control group and 45 kidney transplant patients.

Data were analyzed using the Statistical Package for Social Sciences version 20.0 program (SPSS, Chicago, IL). Normality of continuous variables was assessed by the Shapiro Wilk test. Data with normal distribution are presented as mean ± standard error, whereas data with asymmetric distribution are presented as median (interquartile range). Body fat and trunk fat assessed by DEXA were compared at three and twelve months in kidney transplant recipients by paired t test. For comparisons between control group and kidney recipients at twelve months after transplantation, Student’s t test or Mann Whitney test were used, as appropriate. Categorical variables were compared among groups by Chi-square test and they are reported as absolute numbers and percentages. Correlations were tested by Pearson’s or Spearman’s correlation coefficient, according to variable distribution. To assess changes over time in kidney transplantation group, Friedman test was used for asymmetric variables. Generalized estimating equations (GEE) with linear model and Bonferroni correction were used when variables presented normal distribution. In GEE multivariable model, serum PGRN was entered as dependent variable, and age, gender and study period were included as constant variables. First, each variable that presented statistical significance over time or correlated to PGRN was tested in this model to identify which one was related to serum PGRN. Then, variables with p<0.10 were included in a multivariable model, where normal distribution of residuals was accepted. Only valid cases were included in each analysis. The level of statistical significance was established at lower than 5%.

## Results

### Clinical characteristics

Sixty-four patients were assessed for eligibility. The exclusions were: eight re-transplantation, five kidney-pancreas transplantation and two transplants in systemic lupus erythematosus recipients. Forty-nine kidney transplant recipients were initially enrolled in the study. Three patients did not reach three months of transplantation with functioning graft and were not included in the analyses. Of the forty-six patients included, two lost their kidney graft and two died before the first year of transplantation.

Kidney transplant recipients and controls had similar age, gender and ethnicity distribution (Table 1). Hypertension had high prevalence in both groups, and diabetes mellitus was present in 19.6% in kidney patients before transplantation and in 28.2% of control patients (p = 0.44). After transplantation, five patients (10.9%) developed new-onset diabetes after transplant and the prevalence of diabetes in the study group at the first year of transplantation was 30.5% (p = 0.99 vs. control group) (Table 1).

**Table 1:** Basal characteristics of the study groups.

	TRANSPLANTATION GROUP (n = 46)	CONTROL GROUP (n = 40)	P value
Age, years	49.2 ± 2.1	51.8 ± 2.0	0.40
Male gender, n (%)	27 (58.7)	23 (57.5)	0.99
White ethnicity, n (%)	30 (65.2)	30 (75.0)	0.36
Hypertension, n (%)	41 (89.1)	35 (87.5)	0.99
Diabetes mellitus, n (%)			
Basal	9 (19.6)	11 (28.2)	0.44
Post-transplant	14 (30.5)		0.99
Primary kidney disease, n (%)			
Unknown	15 (32.6)		
Hypertension	10 (21.7)		
Diabetes mellitus	8 (17.4)		
Glomerulonephritis	5 (10.9)		
Polycystic kidney disease	5 (10.9)		
Others	3 (6.5)		
Renal replacement therapy, n (%)			
Hemodialysis	42 (91.3)		
Hemodialysis and peritoneal dialysis	2 (4.3)		
Preemptive transplantation	2 (4.3)		
Dialysis duration before transplantation, months	24 (14.5–52.5)		
Donor type, deceased, n (%)	36 (78.3)		

Among the study group, most patients had unknown primary kidney disease (32.6%), followed by hypertension (21.7%), diabetes mellitus (17.4%), glomerulonephritis (10.9%), adult polycystic kidney disease (10.9%), and other conditions (6.5%) (Table 1). Before transplantation, most patients were on hemodialysis (91.3%) and the median dialysis duration was 24 (14.5–52.5) months. Most patients (78.3%) received their kidney from deceased donors (Table 1).

Induction immunosuppression was employed in 82.6% of patients, basiliximab was used for 10 patients (21.7%) and antithymocyte globulin (ATG) for 28 (60.9%). Maintenance immunosuppression was achieved with prednisone, tacrolimus and mycophenolate for 97.8% of the transplant recipients. One patient (2.2%) received cyclosporine instead of tacrolimus. The cumulative per patient prednisone dose at the end of the first year after transplantation was 3.35 (3.29–3.46) g.

### Laboratorial, anthropometric and body composition characteristics

Laboratory tests, anthropometric measurements and body composition variables are presented in Table 2. As expected, renal function improved after transplantation at 12 months and was similar to control group at this time. Except for IL-6, that was higher in the kidney recipients, no other studied variable presented statistically significant differences between groups.

**Table 2:** Laboratorial, anthropometric and body composition characteristics of kidney transplant recipients and control group.

	TRANSPLANTATION GROUP				CONTROL GROUP (n = 40)	P value* (between groups)
	Before transplant (n = 46)	3 months (n = 46)	12 months (n = 42)	P value (over time)		
eGFR (mL/min/1.73m^2^)	7.87 ± 0.42 ^a^	49.3 ± 2.9 ^b^	56.7 ± 3.7 ^c^	<0.01	58.6 ± 2.9	0.77
IL-6 (pg/mL)	4.46 (3.12–6.92)	3.12 (3.12–5.87)	4.41 (3.12–7.54)	0.32	3.12 (3.12–4.03)	<0.01
hsCRP (mg/dL)	4.43 (2.14–10.31) ^a^	2.20 (0.92–5.05) ^b^	3.19 (1.33–6.37) ^ab^	0.02	4.63 (1.60–9.48)	0.43
FPG (mg/dL)	91.0 (83.0–98.3) ^a^	101.0 (86.8–115.0) ^b^	97.0 (86.0–108.5) ^ab^	0.01	93.0 (83.5–110.3)	0.57
Insulin (mg/dL)	7.60 (4.75–14.30)	9.85 (6.93–13.03)	8.90 (6.40–13.73)	0.07	9.85 (6.58–13.70)	0.50
HOMA	1.73 (0.98–3.31)	2.53 (1.63–3.55)	2.29 (1.34–3.83)	0.10	2.63 (1.52–3.77)	0.70
Total cholesterol (mg/dL)	165.6 ± 5.7 ^a^	188.9 ± 6.8 ^b^	179.9 ± 6.0 ^ab^	0.01	184.1 ± 8.3	0.70
HDL-cholesterol (mg/dL)	35.3 ± 2.0 ^a^	46.3 ± 2.1 ^b^	46.3 ± 2.3 ^b^	<0.01	44.2 ± 1.8	0.47
LDL-cholesterol (mg/dL)	92.9 ± 4.2	102.0 ± 5.1	96.8 ± 4.5	0.23	109.4 ± 7.1	0.12
Triglycerides (mg/dL)	158.0 (108.0–226.8)	183.0 (126.8–232.3)	148.5 (111.5–202.0)	0.26	136.5 (88.5–213.3)	0.22
Body mass index (kg/m^2^)	27.3 ± 0.7 ^ab^	26.7 ± 0.7 ^a^	27.9 ± 0.8 ^b^	<0.01	29.5 ± 0.80	0.22
Waist circumference (cm)	93.3 ± 2.7 ^ab^	93.8 ± 1.7 ^a^	96.3 ± 2.0 ^b^	0.01	99.9 ± 2.3	0.26
Bioimpedance body fat (%)	26.5 ± 1.5	27.5 ± 1.2	28.0 ± 1.3	0.40	27.9 ± 7.4	0.91
DEXA body fat (%)	-	33.2 ± 1.4	34.3 ± 1.4	0.06	35.2 ± 1.3	0.67
DEXA trunk fat (kg)	-	13.8 ± 0.9	14.8 ± 1.0	0.02	16.7 ± 1.1	0.19

eGFR: estimated glomerular filtration rate; IL-6: interleukin-6; hsCRP: high sensitivity C reactive protein; FPG: fasting plasma glucose; HOMA: Homeostasis Model Assessment; DEXA: Dual-energy X-ray absorptiometry.

* Control group was compared to transplantation group at 12 months.

In the transplant group, changes in hsCRP, FPG, total cholesterol, HDL-cholesterol, BMI and waist circumference were observed over time (Table 2). In the early period after transplantation, there was a significant reduction in the hsCRP and increment in the FPG, total cholesterol and HDL-cholesterol levels. At one year after transplantation, there was a significant increase in BMI and waist circumference compared to immediate period after transplantation. There was also a gain in trunk fat during this period (Table 2).

**Fig 1:**
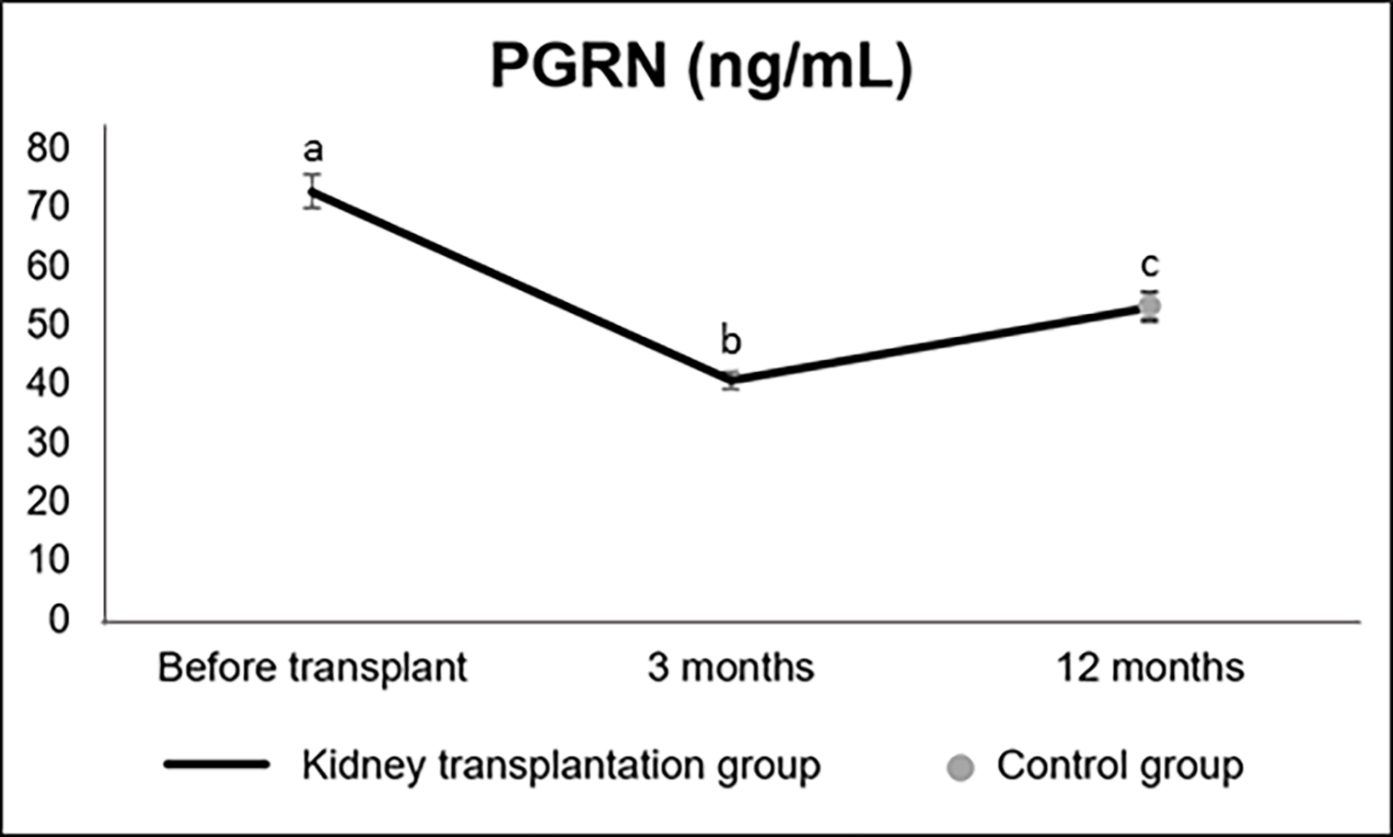
Serum PGRN up to 12 months after transplantation and in control group (mean ± standard error). PGRN: progranulin. Significant differences in the kidney transplant group are presented by letters (a, b, c).

Serum PGRN significantly decreased in the early period after transplantation (from 72.78 ± 2.86 ng/mL before transplantation to 40.65 ± 1.49 ng/mL at three months; p<0.01) and increased at one year (53.15 ± 2.55 ng/mL; p<0.01 vs. three months), remaining significantly lower than before transplantation (p<0.01) (p_over time_<0.01). At 12 months, PGRN value was similar to controls (53.31 ± 2.11 ng/mL; p = 0.97) (Fig 1).

**Fig 2:**
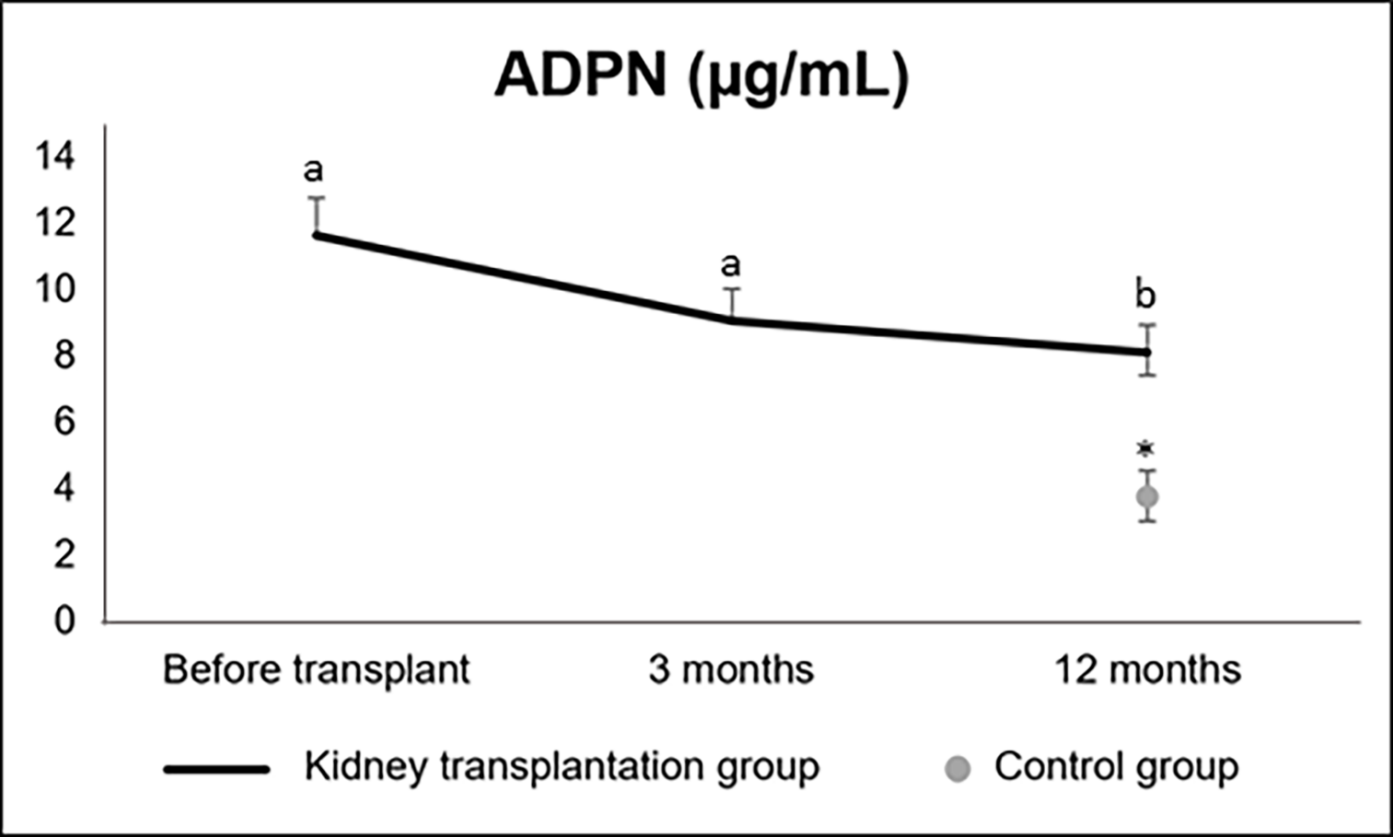
Serum ADPN up to 12 months after transplantation and control group (median ± standard error). ADPN: adiponectin. Significant differences in the kidney transplant group are presented by letters (a, b, c). * Significant difference between groups.

Serum ADPN had a not-significant drop at three months [from 11.64 (7.74-22.55) μg/mL before transplantation to 9.08 (7.16-17.63) μg/mL at three months; p = 0.38] and further dropped at twelve months [8.10 (6.06-11.82) μg/mL; p<0.01 vs. pre-transplant and p = 0.04 vs. three months; p_over time_<0.01]. At one year after transplantation, ADPN levels were significantly higher in kidney transplant recipients as compared to controls [3.71 (2.55-6.04) μg/mL; p<0.01) (Fig 2).

### Correlations

Correlations between serum PGRN levels and eGFR, anthropometric and laboratory variables in kidney transplant recipients are presented in Table 3. A positive and significant correlation was observed between serum PGRN and IL-6 before kidney transplantation (r = 0.413; p = 0.01) and at three months after transplant (r = 0.328; p = 0.03) (Table 3). No other correlations were observed.

**Table 3:** Correlations between serum PGRN and laboratory and anthropometric variables in kidney transplant recipients.

	PGRN		
	Before transplant r (p)	3 months r (p)	12 months r (p)
eGFR	-0.244 (0.10)	-0.215 (0.15)	-0.297 (0.06)
Adiponectin	0.215 (0.15)	-0.028 (0.86)	0.007 (0.97)
Interleukin-6	0.413 (0.01)	0.328 (0.03)	0.282 (0.07)
High sensitivity C reactive protein	0.191 (0.22)	0.192 (0.20)	0.200 (0.21)
Fasting plasma glucose	-0.142 (0.35)	0.190 (0.21)	0.180 (0.26)
HOMA	-0.154 (0.32)	0.223 (0.14)	0.160 (0.32)
Body mass index	-0.006 (0.97)	0.066 (0.66)	-0.023 (0.89)
Waist circumference	0.116 (0.44)	0.225 (0.13)	0.203 (0.20)
Bioimpedance body fat	-0.229 (0.14)	0.056 (0.71)	-0.056 (0.73)
DEXA body fat	-	-0.029 (0.85)	0.074 (0.64)
DEXA trunk fat	-	0.072 (0.63)	0.066 (0.68)

PGRN: progranulin; eGFR: estimated glomerular filtration rate; HOMA: Homeostasis Model Assessment; DEXA: Dual-energy X-ray absorptiometry.

### Multivariable analysis

Waist circumference, adiponectin and hsCRP showed significant association in the univariable analyses adjusted for age, gender and study period, using serum PGRN as dependent variable (Table 4). In the GEE multivariable model, waist circumference remained independently associated to serum PGRN levels, and FPG also showed a significant association (Table 4).

**Table 4:** Generalized estimating equations multivariable model using serum PGRN as dependent variable.

Variable	Beta	95% Confidence Interval	P value
Univariable*			
eGFR	-0.153	-0.317 to 0.012	0.07
Interleukin-6	0.810	-0.049 to 1.669	0.06
High sensitivity C reactive protein	0.468	0.281 to 0.654	<0.01
Fasting plasma glucose	0.032	-0.001 to 0.066	0.06
Total cholesterol	-0.030	-0.094 to 0.033	0.35
HDL cholesterol	-0.198	-0.515 to 0.119	0.22
Body mass index	-0.045	-0.736 to 0.646	0.90
Waist circumference	0.116	0.009 to 0.223	0.03
Adiponectin	0.597	0.012 to 1.181	0.04
Multivariable*			
eGFR	-0.067	-0.177 to 0.043	0.23
Interleukin-6	0.417	-0.289 to 1.123	0.25
High sensitivity C reactive protein	0.078	-0.244 to 0.400	0.64
Fasting plasma glucose	0.036	0.005 to 0.067	0.02
Waist circumference	0.152	0.030 to 0.275	0.01
Adiponectin	0.551	-0.047 to 1.150	0.07

* Adjusted for age, gender and study period.

PGRN: progranulin; eGFR: estimated glomerular filtration rate.

## Discussion

In this longitudinal study, it was possible to demonstrate changes in PGRN serum concentration overtime after kidney transplantation. In patients with end-stage renal disease, PGRN is elevated, and it decreases upon eGFR improvement in the early period after kidney transplantation. At one year, an increment in PGRN is observed, seems to be independent of eGFR, and remained significantly lower than before transplantation.

The reduction in serum PGRN observed immediately after transplantation could be attributed to improvement in kidney function. To our knowledge, this is the first study evaluating the effect of kidney transplantation (and consequently greater eGFR) in PGRN levels. It seems that renal clearance is an important route of PGRN elimination. Some studies have demonstrate that patients with impaired renal function have increased PGRN circulating levels [11–13]. This is observed both in diabetic and non-diabetic kidney disease humans [11–13] and also in a mice model of CKD [25]. Moreover, some patients who present higher PGRN serum levels have lower urinary excretion [11]. These findings are consistent with the PGRN kinetics reported in the present study. Although evidence suggests the eGFR as an independent predictor of serum PGRN [12], we found other variables involved in the PGRN levels.

Weight gain is common following kidney transplantation. It occurs mainly due to increased appetite and freedom of dietary restrictions needed during the dialysis period [14, 26, 27]. In this study, kidney transplant recipients had increased BMI, waist circumference and trunk fat at one year after transplantation compared to three months. It was previously reported that these conditions are associated with higher serum PGRN [8–10]. Qu et al. [10] compared serum PGRN in obese and non-obese patients without kidney disease and found higher levels in obese individuals. Youn et al. [8] investigated the relationship between PGRN and visceral adiposity. They found that patients with a predominantly visceral fat distribution had significantly higher PGRN serum levels [8]. In agreement, we found waist circumference independently associated to circulating PGRN in multivariable analysis. Previous studies show that PGRN has a positive correlation with BMI [8, 10, 11, 28], BF% and waist circumference [8, 9, 11, 28, 29].

We observed a significant correlation between serum PGRN and IL-6 before and in the early period after transplantation. That comes in support to previous studies [10, 30] and suggests an important relationship between these markers pointing to PGRN as a pro-inflammatory molecule. Matsubara et al. [5] demonstrate that PGRN promotes IL-6 expression in adipose cells, which impairs insulin signaling. This adipokine has also chemotactic activity, recruiting monocytes into adipose tissue [8]. Moreover, PGRN binds to the tumor necrosis factor receptor 1 (TNFR-1) [28, 31] and experimentally induces adipose insulin resistance [32]. In humans, increased PGRN serum levels have been found in individuals with insulin resistance [33] and T2DM [6, 8–10]. In the present study, FPG was associated to serum PGRN levels in the multivariable model.

PGRN metabolic functions are not fully understood. Some authors have reported that binding PGRN to TNFR-1 could impair tumor necrosis factor alfa (TNF-α) binding to its receptor, resulting in an anti-inflammatory effect [34, 35]. This hypothesis is also supported in a mice model of renal ischemia-reperfusion injury, where PGRN deficiency was associated with higher elevation of serum creatinine and blood urea nitrogen and its administration in vitro attenuated inflammation [36]. Similarly, PGRN-deficient mice exposed to lipopolysaccharide (LPS) injection as an endotoxin-induced acute kidney injury model, presented increments of inflammatory markers, serum creatinine and blood urea nitrogen [37]. Moreover, administration of recombinant PGRN before LPS treatment in wild-type mice was associated with reduced renal injury [37]. Finally, in a mice model of hyperhomocysteinemia (a risk factor for kidney disease), PGRN-deficient mice also presented exacerbated renal injury, that could be ameliorated by pretreatment with recombinant human PGRN [38]. In this context, PGRN could be a renal protective molecule in an inflammatory environment.

ADPN is a well-described anti-inflammatory adipokine, that is frequently reduced in obesity and insulin resistance conditions [39]. In CKD, ADPN levels have been reported to be increased [18, 40, 41]. This is in agreement with our findings before transplantation. It was previously described that the adipose tissue production of ADPN is increased in end-stage renal disease, contributing for its increased circulating levels [42]. The beneficial effects of higher ADPN, however, are not effective in CKD, mainly due to ADPN resistance at the post-receptor level [43]. In the present work, ADPN concentration decreased at three months and the decrement reached statistical significance at one year, findings that are corroborated by previous studies [17, 18, 41]. This reduction could be associated with improved eGFR and other factors as increased in BMI, waist circumference and trunk fat [18, 39]. We further observed that serum ADPN was increased in renal recipients compared to controls, which is also in accordance with other studies [17, 44, 45]. No correlation between PGRN and ADPN was observed, except in the univariable analysis. A finding corroborated by many [8, 30, 46], but not all [12] studies.

Other adipokines, mainly leptin, were previously evaluated in kidney transplant recipients [17]. Serum leptin levels evaluated up to five years after renal transplantation [14] presented a similar behavior to PGRN: elevated levels pre-transplantation, decline in the early period after transplantation and later increment [14, 47]. We believe that the reasons for such behaviors are similar: improvement in eGFR, followed by metabolic changes and weight gain occurring after kidney transplantation [14, 47].

This study provides relevant information regarding the effects of kidney transplantation on PGRN serum levels. However, there are some limitations. First, follow-up is relatively short and longer evaluation is needed to a better understanding of the relationships between PGRN and other parameters in kidney transplantation. Second, bioelectric impedance and DEXA methods have limitations in estimating body fat in kidney graft recipients [48]; and parameters derived from these methods might be seeing cautiously.

In conclusion, PGRN serum levels are increased before transplantation and a reduction is observed in the early period after transplantation, possibly attributed to an improvement in the renal function. At later period of transplantation, an increment in PGRN is observed, and seems to be related to metabolic profile changes. Further studies are needed to better understand these complex relationships of PGRN in this population.
